# *BRMS1* gene expression may be associated with clinico-pathological features of breast cancer

**DOI:** 10.1042/BSR20170672

**Published:** 2017-08-21

**Authors:** Li-Zhong Lin, Miao-Guo Cai, Yue-Chu Dai, Zhi-Bao Zheng, Fang-Fang Jiang, Li-Li Shi, Yin Pan, Han-Bing Song

**Affiliations:** 1 Department of Oncology, Taizhou Central Hospital, Taizhou 318000, P.R. China; 2 Department of Oncology, Luqiao Branch of Taizhou Hospital, Taizhou 318000, P.R. China; 3 Department of Infection, Luqiao Branch of Taizhou Hospital, Taizhou 318000, P.R. China

**Keywords:** BRMS1, Breast cancer, Clinico-pathological features, Meta-analysis, Metastasis, Prognosis

## Abstract

Our aim is to investigate whether or not the breast cancer metastasis suppressor 1 (*BRMS1*) gene expression is directly linked to clinico-pathological features of breast cancer. Following a stringent inclusion and exclusion criteria, case–control studies with associations between *BRMS1* and breast cancer were selected from articles obtained by way of searches conducted through an electronic database. All statistical analyses were performed with Stata 12.0 (Stata Corp, College Station, TX, U.S.A.). Ultimately, 1,263 patients with breast cancer were found in a meta-analysis retrieved from a total that included 12 studies. Results of our meta-analysis suggested that BRMS1 protein in breast cancer tissues was significantly lower in comparison with normal breast tissues (odds ratio, OR = 0.08, 95% confidence interval (CI) = 0.04–0.15). The BRMS1 protein in metastatic breast cancer tissue was decreased than from that was found in non-metastatic breast cancer tissue (OR = 0.20, 95%CI = 0.13–0.29), and BRMS1 protein in tumor-node-metastasis (TNM) stages 1 and 2 was found to be higher than TNM stages 3 and 4 (OR = 4.62, 95%CI = 2.77–7.70). BRMS1 protein in all three major types of breast cancer was lower than that of control tissues respectively. We also found strong correlations between BRMS1 mRNA levels and TNM stage and tumor size. The results our meta-analysis showed that reduction in *BRMS1* expression level was linked directly to clinico-pathological features of breast cancer significantly; therefore, suggesting the loss of expression or reduced levels of *BRMS1* is potentially a strong indicator of the metastatic capacity of breast cancer with poor prognosis.

## Introduction

Breast cancer accounts for approximately 33% of cancers diagnosed among females in the U.S.A., and is the second leading cause of cancer deaths worldwide [1]. Generally, older women are more commonly diagnosed with breast cancer, with one in 200 women before the age of 40 being diagnosed [2]. Generally, approximately 40% of all patients with breast cancer experience a relapse, of which recurrences 10–20% are locally metastatic and 60–70% are distant metastases [3]. The established risk factors for breast cancer are age, menopausal status, body mass index (BMI), duration of breastfeeding, age at first pregnancy, and postmenopausal hormonal use [4–6]. The predominant cause of death in breast cancer is distant metastasis [7]. Tumor metastasis is a multistep process involving disruption of intercellular adhesions and dispersal of single cells from solid tumor, invasion of blood and lymphatic vessels, immunologic escape in circulation, attachment to endothelial cells, extravasation from blood and lymph vessels and proliferation, and angiogenesis induction [8,9]. This process is assisted by a preferred microenvironment at the primary and metastatic sites [7].

Breast cancer metastasis suppressor 1 (BRMS1), introduced in 2000, inhibits metastasis while having no effect on the growth of primary tumors [10]. The *BRMS1* gene is located at 11q13.1-13.2, a region often altered in late-stage breast cancers, while in close proximity to the genomic loci that contains deletions and amplifications commonly observed in progression of breast cancer [11]. BRMS1 is also an inhibitor of metastasis in ovarian cancer, bladder cancer, non-small cell lung cancer, melanoma, and breast cancer. Metastasis mouse models have demonstrated a high capacity for BRMS1 to inhibit metastasis, recording up to 80–90% metastasis inhibition [8,12,13]. The mechanisms by which *BRMS1* mediates its anti-metastasis function still remain unknown despite observations of anti-metastatic potential of BRMS1 [7]. Following this context, it is also important that thorough evaluation of the BRMS1 association with metastatic breast cancer is performed, so for this reason performing a meta-analysis based study to test the correlation between *BRMS1* expression and the tumor behavior in breast cancers alone was necessary.

## Materials and methods

### Search strategy

A literature search was performed systematically using PubMed, EBSCO, SpringerLink, Wiley, Ovid, Web of science, Wanfang Database, China National Knowledge Infrastructure (CNKI), and VIP Information databases using MeSH and free text search terms (last updated search on October, 2014). All variants of key search terms: breast cancer and *BRMS1* were included. For example, (‘BRMS1 protein, human’ or ‘breast-cancer metastasis suppressor 1’) and (‘breast neoplasms’ or ‘breast cancer’ or ‘breast carcinoma’ or ‘tumors, breast’ or ‘mammary neoplasms, human’ or ‘carcinoma, human mammary’ or ‘mammary cancer’ or ‘malignant neoplasm of breast’ or ‘malignant tumor of breast’ or ‘cancer of the breast’) were selected to retrieve corresponding literatures. Bibliographies regarding the collected trials and review papers were studied and explored manually for potentially relevant and conducive articles.

### Criteria for selecting articles included in this meta-analysis

Study articles were incorporated if (1) the study type of selected studies was case–control study; (2) study subjects were patients diagnosed with breast cancer and healthy controls; (3) selected studies provided complete data consisting of sample size, age, ethnicity, gender, pathological types, positive expression rate of BRMS1 protein, expression of BRMS1 mRNA etc.; (4) the extracted studies were published by the same authors, only including the last or complete one.

Studies were omitted if they were unrelated with either *BRMS1* expression or breast cancer, the data were incomplete, the study was not published in Chinese and English, or if the article was repeatedly published.

### Data extraction

Information was extracted from all included publications systematically by two investigators in adherence with the aforementioned inclusion criteria. The following data were collected from each individual study: first author, country, language, ethnicity, study design, total numbers and mean age of cases and controls, sample size, pathological types etc.

### Statistical analysis

All of the meta-analyses performed utilized Stata 12.0 (Stata Corp, College Station, TX, U.S.A.). The standardized mean differences (SMDs), odds ratio (OR), and effect size (ES) with 95% confidence interval (CI) were all used to assess the case–control studies investigating the association between BRMS1 and clinico-pathological features of breast cancer. Moreover, *Z*-test was applied to determine the significance of pooled SMDs. Cochran’s Q statistic with a significance level of *P* < 0.05 and the *I*^2^ test (0–100%, values of 40% and 75% were considered to indicate moderate and high heterogeneity respectively) were used to assess heterogeneity across studies. If *P* < 0.05 or *I*^2^ > 50%, there was great heterogeneity among studies, thereby implementing use of a random effect model; if no presence of a random effect model, a fixed effect model was performed [14,15].

## Results

### Literature searching results and baseline characteristics of included studies

Originally through database searches, one hundred and seventy-five articles were identified. Thirty seven papers remained after excluding duplicates (*n* = 15), animal studies (*n* = 24), letters, reviews, meta-analyses (*n* = 2), and unrelated topics (*n* = 97). After excluding non-case–control or cohort study (*n* = 11), studies not relevant to *BRMS1* (*n* = 6), studies with no correlation to breast cancer (*n* = 7), insufficient information in studies (*n* = 1), 12 articles were finally selected for this meta-analysis [16–27], including 1,263 patients with breast cancer. Among the 12 studies, there were study subjects with nine being performed in Asian trials, two trials in Caucasians, and one trial in Mixed. To break it down further, in compliance with country there were eight studies from China, one from Japan, U.S.A., England, and Italy respectively. The included studies were all published between 2006 and 2014. *BRMS1* in different breast tissues, lymph node metastasis (LNM) status, tumor-node-metastasis (TNM) stages, tumor size, histological grades, pathological types, estrogen receptor (ER) status, progesterone receptor (PR) status, overall survival (OS), and relapse free survival (RFS) expressions were all compared in this meta-analysis. The sample size of study subjects ranges from 70 to 200. The marker involved in these studies was either protein or mRNA. The baseline characteristics of included studies are shown in Table 1 respectively.

**Table 1:** Baseline characteristics of included studies

Author	Year	Country	Ethnicity	Language	Disease	Age (years)	Marker	Sample number
Wang, D.Y. [16]	2014	China	Asians	Chinese	Breast neoplasms	44.5(28-78)	Protein	160
Wang, L.X. [17]	2013	China	Asians	Chinese	Breast neoplasms	52.0(37-76)	Protein	65
Han, D.Y. [18]	2012	China	Asians	Chinese	Breast neoplasms	52.0(33-86)	Protein	75
Wu, Z.Y. [19]	2011	China	Asians	Chinese	Breast neoplasms	49.4(32-74)	Protein	63
He, X.B. [20]	2009	China	Asians	Chinese	Breast neoplasms	46.2(27-75)	Protein	78
Cui, M. [22]	2009	China	Asians	Chinese	Breast neoplasms	45.7(29-76)	Protein	80
Frolova, N. [21]	2009	U.S.A.	Mixed	English	Breast neoplasms	25-89	mRNA	174
Tang, L.B. [23]	2007	China	Asians	Chinese	Breast neoplasms	52.4(28-83)	mRNA	71
Lombardi, G. [24]	2007	Italy	Caucasians	English	Breast neoplasms	NR	mRNA	47
Zhang, Y.L. [26]	2006	China	Asians	Chinese	Breast neoplasms	43.5(31-62)	mRNA	51
Zhang, Z.H. [25]	2006	Japan	Asians	English	Breast neoplasms	53.0(34-88)	mRNA	161
Hicks, D.G. [27]	2006	U.K.	Caucasians	English	Breast neoplasms	NR	mRNA	238

NR, not reported.

### Breast cancer tissues and normal tissues

Administration of a heterogeneity test discovered the lack of heterogeneity in the expression of BRMS1 protein between breast cancer tissues and normal tissues, requiring implementation of a random effect model (*P* = 0.219, *I*^2^ = 32.1%). There was heterogeneity in expression of BRMS1 mRNA between breast cancer and normal tissues, thus a fixed-effect model was performed (*P* < 0.001, *I*^2^ = 99.5%).

**Figure 1:**
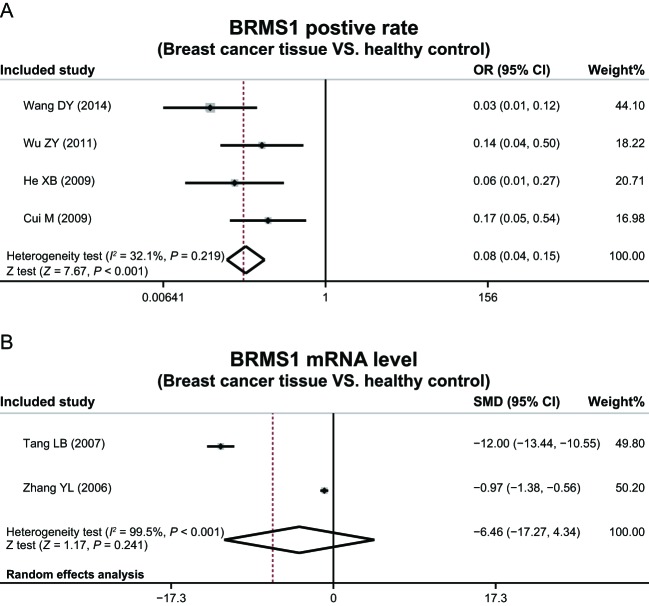
The expressions of *BRMS1* in breast cancer and healthy tissues.

Results of this meta-analysis suggested that the expression of BRMS1 protein in breast cancer tissues was significantly lower in comparison with normal tissues (OR = 0.08, 95%CI = 0.04–0.15, *P* < 0.001), while showing no significant difference in expression of BRMS1 mRNA between breast cancer and normal tissues (OR = −6.46, 95%CI = −17.27–4.34, *P* = 0.241) (Figure 1).

### Clinico-pathological features of breast cancer

The heterogeneity test revealed absence of heterogeneity in expression of BRMS1 protein in different LNM status, TNM stages, tumor size, histological grades, pathological types, therefore leading to adoption of a fixed-effect model (LNM: *P* = 0.229, *I*^ 2^ = 27.5%; TNM stage: *P* = 0.892, *I*^2^ = 0.00%; tumor size: *P* = 0.348, *I*^2^ = 10.2%; histological grade: *P* = 0.998, *I*^2^ = 0.00%; ductal carcinoma: *P* = 0.466, *I*^2^ = 0.00%; lobular carcinoma: *P* = 0.473, *I*^2^ = 0.00%; medullary carcinoma: *P* = 0.653, *I*^2^ = 0.00%) simulating heterogeneity. There was heterogeneity in expression of *BRMS1* mRNA in LNM and TNM stage, thus random-effects model was performed (LNM: *P* < 0.001, *I*^2^ = 97.2%; TNM stage: *P* = 0.003, *I*^2^ = 88.7%), while showing no heterogeneity in tumor size, and thereby requiring use of a fixed-effect model (*P* = 0.697, *I*^2^ = 0%).

**Figure 2:**
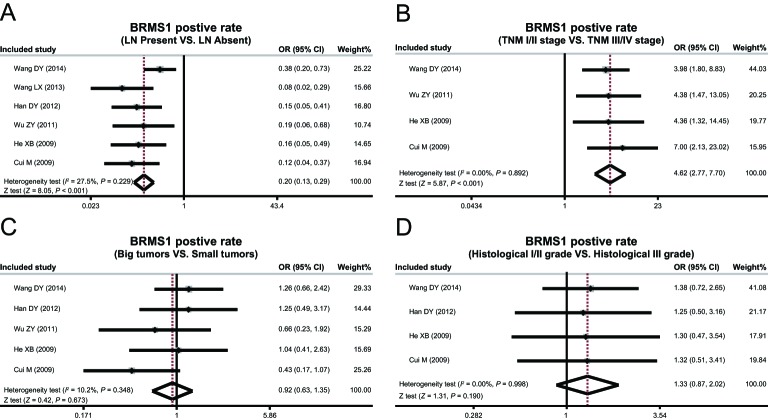
Positive expression rates of BRMS1 protein in different LNM status, tumor size, and histological grades of breast cancer.

**Figure 3:**
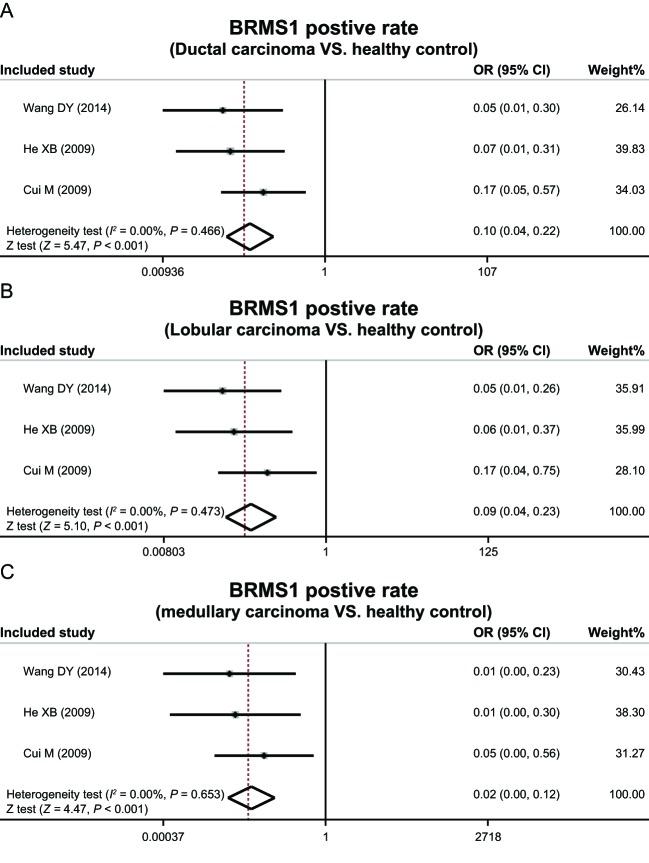
Positive expression rates of BRMS1 protein in different pathological types of breast cancer.

The expression of BRMS1 protein in metastatic breast cancer tissue was found to be lower than that of non-metastatic breast cancer tissue (OR = 0.20, 95%CI = 0.13–0.29, *P*<0.001), and the expression of BRMS1 protein in TNM stages 1 and 2 was higher than TNM stages 3 and 4 (OR = 4.62, 95%CI = 2.77–7.70, *P*<0.001) by means of the meta-analysis used. In addition, BRMS1 protein expression level was not considerably related with tumor size and histological grade (tumor size: OR = 0.92, 95%CI = 0.63–1.35, *P* = 0.673; histological grade: OR = 1.33, 95%CI = 0.87–2.02, *P* = 0.190) (Figure 2). Additionally, expression of BRMS1 protein in the three types of breast cancer tissues was all lower than the normal tissues (ductal carcinoma: OR = 0.10, 95%CI = 0.04–0.22, *P* < 0.001; lobular carcinoma: OR = 0.09, 95%CI = 0.04–0.23; *P* < 0.001; medullary carcinoma: OR = 0.02, 95%CI = 0.00–0.12, *P* < 0.001) (Figure 3).

**Figure 4:**
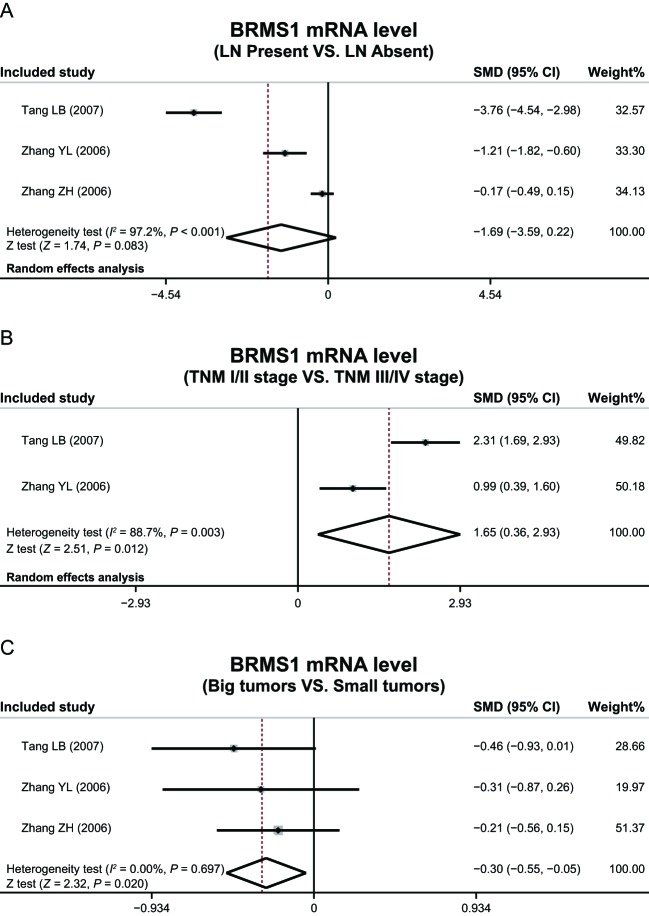
Expression levels of *BRMS1* mRNA in different LNM status, tumor node metastases stages, and tumor size.

This meta-analysis also indicated that expression of BRMS1 mRNA was adversely associated with TNM stage and tumor size (TNM stage: OR = 1.65, 95%CI = 0.36–2.93, *P*=0.012; tumor size: OR = −0.30, 95%CI = −0.55–0.05, *P*=0.020), while association with LNM status (OR = −1.69, 95%CI = −3.59–0.22, *P*=0.083) (Figure 4) proved nonexistent.

### Immunohistochemistry

**Figure 5:**
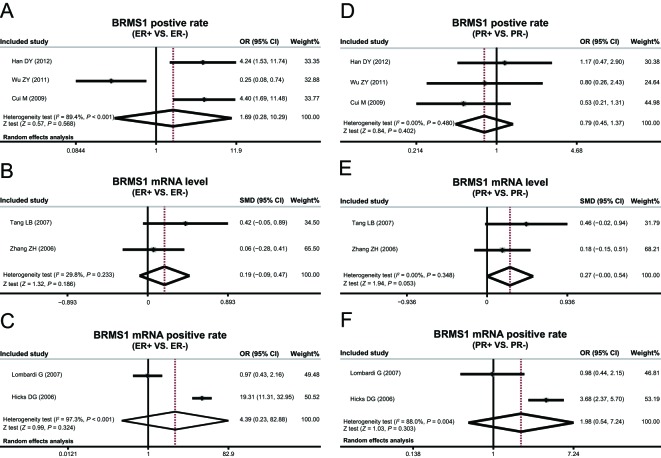
Correlation of *BRMS1* expressions with ER and PR.

Since heterogeneity was nonexistent among studies exploring the correlation between BRMS1 protein expression and PR status, a fixed-effect model was used (*P* = 0.480, *I*^2^ = 0.00%) substituting the lack thereof. However, with the presence of heterogeneity among studies investigating the associations of BRMS1 protein expression and ER status, application of a random-effect model (*P* < 0.001, *I*^2^ = 89.4%) was allowed. Furthermore, no heterogeneity existed across studies investigating the links between the expression of BRMS1 mRNA with status of ER and PR, and again due to the absence of heterogeneity, a fixed-effect model was put in place (ER: *P* = 0.233, *I*^2^ = 29.8%; PR: *P* = 0.348, *I*^2^ = 0.00%), while heterogeneity existed among studies relative to positive expression rate of BRMS1 mRNA and ER and PR status, a random-effect model was utilized (ER: *P* < 0.001, *I*^2^ = 97.3%; PR: *P* = 0.004, *I*^2^ = 88.0%). The expressions of BRMS1 protein and *BRMS1* mRNA were not significant in coordinance with the status of ER and PR in association with the results of this meta-analysis (as shown in Figure 5). The correlation also suggests which test model will be implemented according to presence of heterogeneity.

### Prognosis

No heterogeneity among studies that investigated the correlation of BRMS1 protein expression with OS and RFS was detected thereby administering performance of a fixed-effect model (OS: *P* = 0.329, *I*^2^ = 0.00%; RFS: *P* = 0.488, *I*^2^ = 0.00%). Had there been heterogeneity, the implication of a random-effect model (ER: *P* < 0.001, *I*^2^ = 97.3%; PR: *P* = 0.004, *I*^2^ = 88.0%) would have been necessary to facilitate lack of expression.

**Figure 6:**
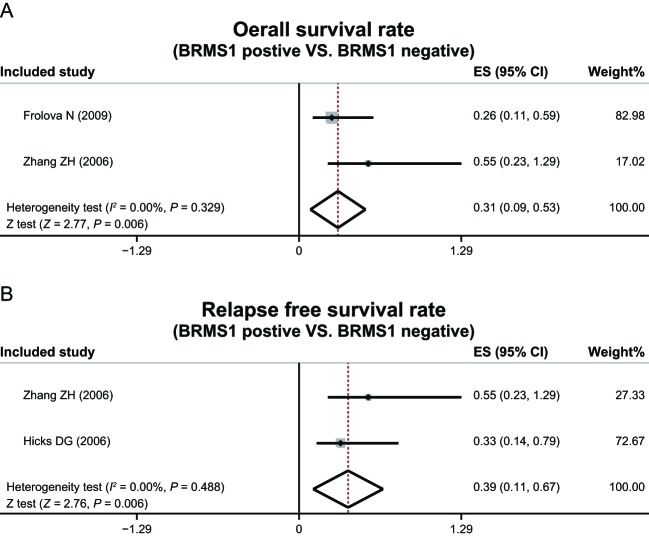
OS rates and RFS rates on the correlation between *BRMS1* and breast cancer.

Specifications that there were significant differences in OS and RFS between patients with positive *BRMS1* (*BRMS1^+^*) and negative *BRMS1* (*BRMS1^−^*) (OS: ES = 0.31, 95%CI = 0.09–0.53, *P*=0.006; RFS: ES = 0.39, 95%CI = 0.11–0.67, *P*=0.006) (Figure 6) were present in this meta-analysis.

## Discussion

The present meta-analysis investigated the correlation between *BRMS1* and the clinico-pathological features of breast cancer including LNM, TNF stages, tumor size, histological grade, pathological type, ER, PR, OS, and RFS. Aside from involving degradation of extracellular matrix, invasion and metastasis of malignant tumors also include reciprocation between various genes such as oncogenes, tumor suppressor genes, and metastasis-regulating genes [28]. Currently, approximately 30 metastasis suppressor genes have been recognized in multiple carcinomas along with the loss of their function through mutations or gene silencing, facilitating metastatic behavior of tumor cells [8]. BRMS1 is an important nuclear protein providing various functions such as differentially modulating the expression of various genes and inhibiting metastasis with no effects on the primary tumor growth, all while regulating migration of tumor cells [29].

In multiple human tumors, there is either a reduction or complete absence of the expression of the BRMS1 nuclear protein. Current study illustrates that BRMS1 protein expression in breast cancer tissue is significantly lesser than normal tissue. In addition to decreased protein expression, the BRMS1 expression in metastatic breast cancer tissue was lower than non-metastatic breast cancer tissue, suggesting invasion and metastasis of breast cancer might possibly be linked to the reduction or absence of *BRMS1* expression. Potential aspects for *BRMS1*-mediated metastasis suppression in breast cancers might be through altering metastasis-associated microRNA and/or interfering with specific cellular pathways relative to metastasis including: gap junctions, nuclear factor kappa B signaling, phosphoinositide signaling, cell motility and invasion, apoptosis, and tumor cell dissemination, though the exact mechanisms remain elucidated [30–36]. Past studies alluded to reports that *BRMS1* reduced lung metastasis in athymic mice when its expression was restored by exogenous expression of BRMS1 in metastatic cell lines from non-small cell lung carcinomas, ovarian, melanoma, and breast [37,38].

In TNM stages 1 and 2, we found that since the expression of BRMS1 protein was drastically higher compared with TNM stages 3 and 4, breast cancer progression might be correlated with low expression of BRMS1 protein. Additionally, another discovery made was finding that the expression levels of BRMS1 mRNA were associated negatively with the TNM stage and tumor size, similar to the result between *BRMS1* expression and TNM staging [39]. Contrary to our result which revealed that the expression levels of BRMS1 mRNA were irrelative to LNM, the BRMS1 mRNA expression levels were found to be lower in brain metastasis of breast cancer than in primary tumor while also reduced in breast tumor compared with the expression of that in matched normal breast tissues [40,41]. Limitations in the present study may have led to possible differences in expression levels. In conclusion, a significant difference in the OS and RFS between patients with *BRMS1*^+^ and *BRMS1*^−^suggests that the BRMS1 protein might be a potential prognostic indicator in breast cancer, which is consistent with a study reported by Hanker et al. which determined that low BRMS1 mRNA expression and poor prognosis of breast cancer were indeed related [42].

Our results might be adversely affected due in part by the small portion size provided by the limitations of the meta-analysis used in this study. Another complication of the limitations of this analysis is the loss of data in several studies, which could have an effect on the final result to a certain extent. Furthermore, our results deemed that there was no association between BRMS1 protein and mRNA expression. The expression of BRMS1 protein in breast cancer tissues was significantly lower compared with normal tissues, while no significant difference in expression of BRMS1 mRNA between breast cancer and normal tissues was found. This was a statistical result and might have been caused by the included studies, those of which might differ in the study of BRMS1 protein and mRNA. According to this, previous clinical studies have revealed that the mRNA and BRMS1 protein expressions were not necessarily conjunctive, and the relative level of mRNA in tumors didn’t necessarily associate with the protein level [21,43,44]. Finally, there is little discussion about the expressions of BRMS1 in in-depth classification of breast cancer, like triple-negative breast cancer (TNBC) and ER+ tumors, a potentially impactful limitation of the analysis that required further investigation.

In summary, the coordinance between reduced or loss of expression of *BRMS1* and clinico-pathological features of breast cancer was significant enough to suggest that *BRMS1* could be an indicator of the metastatic capacity and low expression of the protein in regards to the poor prognosis surrounding breast cancer.

## Abbreviations

BMI: body mass index
BRMS1: breast cancer metastasis suppressor 1
CI: confidence interval
ER: estrogen receptor
ES: effect size
LNM: lymph node metastasis
OR: odds ratio
OS: overall survival
PR: progesterone receptor
RFS: relapse free survival
SMD: standardized mean difference
TNBC: Triple-negative breast cancer
TNM: tumor-node-metastasis
